# CSF and venous blood flow from childhood to adulthood studied by real-time phase-contrast MRI

**DOI:** 10.1007/s00381-024-06275-1

**Published:** 2024-01-11

**Authors:** Prativa Sahoo, Jost M. Kollmeier, Nora Wenkel, Simon Badura, Jutta Gärtner, Jens Frahm, Steffi Dreha-Kulaczewski

**Affiliations:** 1 Department of Pediatrics and Adolescent Medicine, Division of Pediatric Neurology, University Medical Center Göttingen, Robert Koch Street 40, 37075 Göttingen, Germany; 2 Biomedical NMR, Max Planck Institute for Multidisciplinary Sciences, Göttingen, Germany

**Keywords:** Real-time phase-contrast MRI, CSF dynamics, Venous flow, Age-related changes of flow, Childhood hydrocephalus

## Abstract

**Purpose:**

In vivo measurements of CSF and venous flow using real-time phase-contrast (RT-PC) MRI facilitate new insights into the dynamics and physiology of both fluid systems. In clinical practice, however, use of RT-PC MRI is still limited. Because many forms of hydrocephalus manifest in infancy and childhood, it is a prerequisite to investigate normal flow parameters during this period to assess pathologies of CSF circulation. This study aims to establish reference values of CSF and venous flow in healthy subjects using RT-PC MRI and to determine their age dependency.

**Methods:**

RT-PC MRI was performed in 44 healthy volunteers (20 females, age 5–40 years). CSF flow was quantified at the aqueduct (Aqd), cervical (C3) and lumbar (L3) spinal levels. Venous flow measurements comprised epidural veins, internal jugular veins and inferior vena cava. Parameters analyzed were peak velocity, net flow, pulsatility, and area of region of interest (ROI). Statistical tests: linear regression, student's t-test and analysis of variance (ANOVA).

**Results:**

In adults volunteers, no significant changes in flow parameters were observed. In contrast, pediatric subjects exhibited a significant age-dependent decrease of CSF net flow and pulsatility in Aqd, C3 and L3. Several venous flow parameters decreased significantly over age at C3 and changed more variably at L3.

**Conclusion:**

Flow parameters varies depending on anatomical location and age. We established changes of brain and spinal fluid dynamics over an age range from 5–40 years. The application of RT-PC MRI in clinical care may improve our understanding of CSF flow pathology in individual patients.

**Supplementary Information:**

The online version contains supplementary material available at 10.1007/s00381-024-06275-1.

## Introduction

Phase-contrast (PC) MRI has long been a valuable tool for investigating cerebrospinal fluid (CSF) flow-related diseases such as various forms of childhood hydrocephalus, Chiari malformations, and spinal cord pathologies as, for example, syringomyelia [1]. Flow-sensitive MRI techniques so far predominantly used cardiac-gated PC MRI which combines data from multiple cardiac cycles to create a cine representation of a single time-averaged cycle [2–4]. Inherently, respiratory changes are neglected. In contrast, real-time phase-contrast (RT-PC) MRI allows analysis of cardio-respiratory coupling with short acquisition times and high spatiotemporal resolution [5–7]. Its independence of any physiological gating enables quantitative flow assessments in real time and therefore reveals both respiratory and cardiac modulations. On these grounds, RT-PC flow MRI has facilitated new insights into CSF and venous blood flow and contributed significantly to our current understanding of their physiology and driving forces [8–10]. Moreover, the findings foster our knowledge of how venous pathologies might link to intracranial pressure dysregulations and related forms of hydrocephalus, a correlation that has long been observed in clinics. However, the routine use of RT-PC MRI in clinical practice is limited up to today. One of the prerequisites to enhance its diagnostic value for the assessment of CSF circulation pathologies is the establishment of normal ranges for flow dynamics and their age-related dependencies.

So far, few studies specifically investigated alterations of CSF flow dynamics in healthy subjects with respect to age and gender [11–13]. These studies have used PC MRI with cardiac gating and focused either on adult subjects with age ranges 20–65 years or on children from 1 month–17 years of age [14–16]. CSF flow was predominantly analyzed in cerebral aqueduct and parameters like peak velocity or stroke volumes were reported to be influenced by age [2, 11–13]. Studies have been performed at cervical spinal level in adult subjects [16, 17]. Though, in the lumbar spine, as per our knowledge, investigations into age-related changes of CSF dynamics are still lacking. However, the lumbar spinal region seems to be tightly correlated to intracranial pressure changes and can be used for monitoring respective alterations in patients [18]. Effects of cardiac pulse as well as of different forms of breathing on thoracal and abdominal pressures and thus CSF dynamics vary along the spinal canal as has previously been shown [19]. These factors might play important roles in disturbed CSF dynamics in spinal cord pathologies in childhood. Furthermore, disorders of CSF circulation have increasingly been linked to venous system pathologies [20–22]. A tight interplay of CSF and venous flow by forced respiration has been revealed in healthy adult populations but not yet in children [10]. Previous studies have also shown flow measurements by RT-PC MRI to be influenced by breathing mode [9, 10]. Following a specific breathing protocol could be difficult in a clinical setting especially in pediatric patients. As our long-term goal is to develop protocols for pediatric patients with CSF disorders, alterations of physiologic flow related to body growth and changes of circulatory conditions need to be established during free breathing.

Pushing towards clinical applications the objectives of this study are (i) to assess CSF and venous flow in the Aqd, cervical and lumber spinal level in healthy subjects from child- to adulthood using RT-PC MRI during free breathing, and (ii) to provide age-dependent ranges for normal CSF and venous flow parameters at these locations.

## Materials and methods

### Subjects

Forty-four healthy volunteers (20 female, 24 male) with age range 5–40 years were recruited with no known illness and contraindication for MRI. MRI data for all subjects were collected retrospectively. The study was approved by the institutional review board. Written informed consent was obtained from each individual and, if applicable, their parents or caregivers.

### Imaging protocol

RT-PC MRI was performed in three transversal cross-sections covering intracranial and spinal CSF at Aqd, cervical spinal level 3 (C3) and lumber spinal level 3 (L3) as well as concomitant venous flow in epidural veins (EV) at C3 and L3, internal jugular vein (IJV) and inferior vena cava (IVC). All subjects were examined in supine position during free breathing. Measurements were performed at 3 Tesla (Magnetom Prisma Fit, Siemens Healthcare, Erlangen, Germany). RT-PC flow MRI was based on highly undersampled radial FLASH sequences with timing-optimized gradient design [23]. Quantitative velocity maps were calculated by a model-based reconstruction technique offering high spatiotemporal resolution and an integrated correction of concomitant magnetic fields [24, 25]. The MRI parameters were set as: repetition time (TR) 5.68 ms, echo time (TE) 4.61 ms, slice thickness 5 mm, flip angle 10°. The field of view was either 192 mm (Aqd, C3) or 256 mm (L3) and the image matrix sizes were adapted (256 and 342) to obtain a fixed in-plane resolution of 0.75 × 0.75 mm^2^. The two flow-encoded datasets were acquired using 11 radial spokes each yielding a temporal resolution of 125 ms per velocity map corresponding to a rate of 8 frames per second. The velocity encoding range (VENC) was adapted to the peak velocities of CSF or blood flow accordingly. While all CSF measurements as well as studies of EV at C3 and L3 exploited low VENC values of 10 to 30 cm s^−1^, measurements of the large veins at C3 and L3 involved higher VENCs of 60 to 100 cm s^−1^. In the aqueduct and at C3 measurements were conducted with a 64-channel head coil, while at L3 suitable elements of an 18-channel thorax coil and a 32-channel spine coil were used.

The flow data of 10 s with free breathing were collected for all subjects and used in further analysis.

### Regions of interest (ROI)

**Fig. 1:**
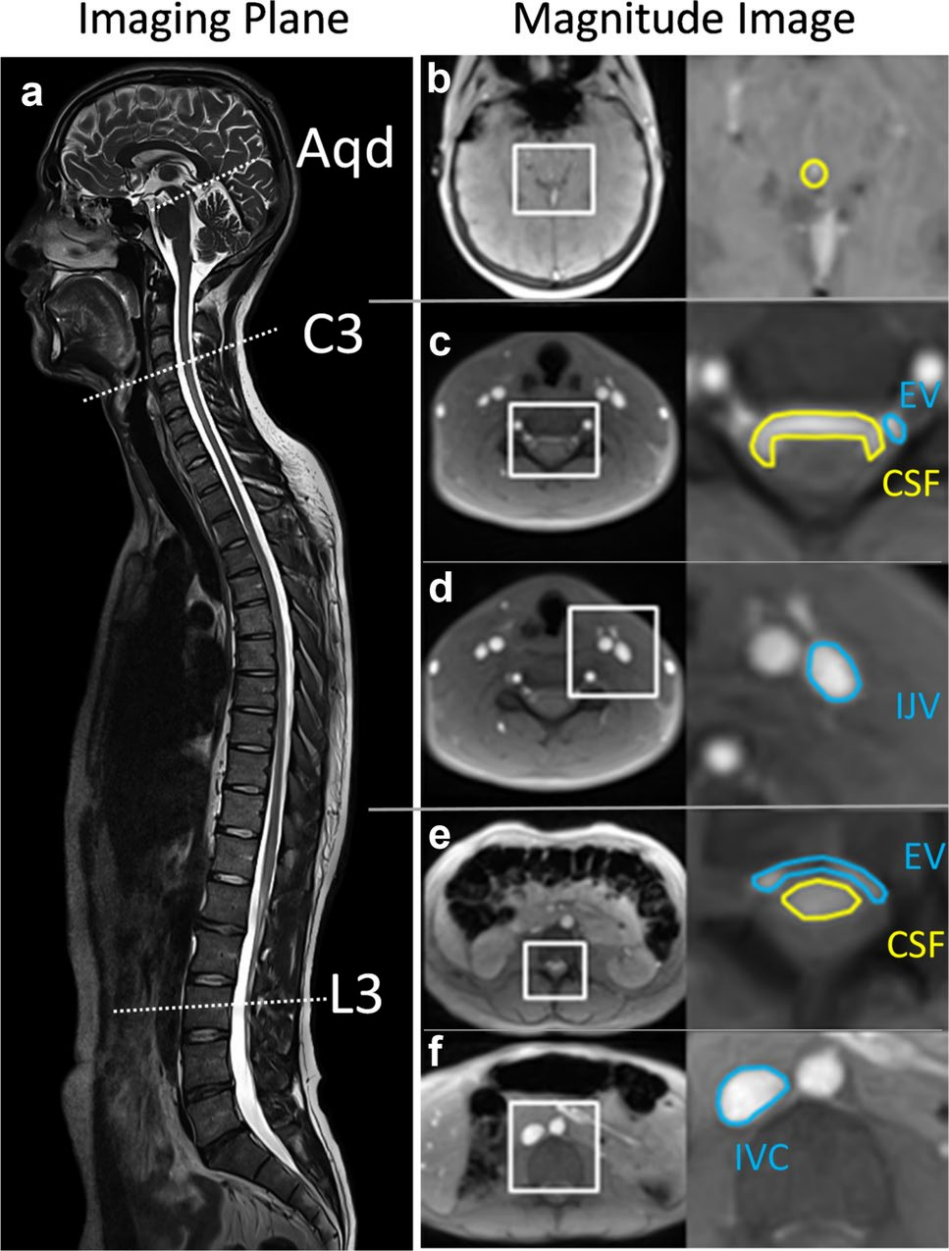
ROIs for CSF and venous flow analyses. **a** The sagittal image indicates the slice locations chosen for Aqd, spinal level C3 and L3. ROIs are shown on axial magnitude images: **b** Aqd, **c** C3 CSF (yellow) and C3 EV (blue), **d** C3 IJV, **e** L3 CSF (yellow) and L3 EV (blue), and **f** L3 IVC. ROI = region of interest, Aqd = aqueduct, C3 = cervical spinal level 3, L3 = lumbar spinal level 3, EV = epidural veins, IJV = internal jugular vein, IVC = inferior vena cava

For the analysis of CSF dynamics ROIs were placed in the Aqd and spinal subarachnoid spaces at C3 and L3 following anatomical borders as close as possible (Fig. 1b–f). For venous flow ROIs were drawn along the vessel walls of the IJV with more pronounced flow signal at C3 (Fig. 1d) and around the IVC at L3 (Fig. 1f). The venous plexus expands in the epidural spaces inside the entire vertebral column (internal vertebral venous plexus) and forms prominent orthogonal veins at C3 known to resemble a rope ladder. The larger one of the two EV was selected for the ROI (Fig. 1c). The lumbar venous plexus creates a spacious mesh with less well identifiable epidural vessels. Here, ROIs were drawn around flow signals detectable in the epidural space ventral to the CSF space (Fig. 1e).

### Data analysis

Real-time flow MRI datasets were quantitatively analysed using CaFuR software (Fraunhofer Mevis, Bremen, Germany) designed to accomplish automatic segmentation of flow signals in real-time image series after manual definition of one initial ROI [26]. The software took care of adjusting the ROI through all dynamic frames. Flow time curves ${F}_{t}$ are computed as the product of mean velocity time curve ${V}_{t}$ within a ROI and the ROI area. Peak velocity (peakVelocity in cm sec^−1^), net flow (netFlow in ml min^−1^), and standard deviation of flow (stdFlow in ml min^−1^) with respect to time were quantified. Standard deviation of flow largely reflects the pulsatility of flow dynamics in a more robust manner than a pulsatility measure that is based on the difference of single noisy minimum and maximum phase values. To illustrate flow components related to respiration and cardiac pulsation, the flow time curve was analysed in the frequency domain. The relative contribution of respiration versus cardiac components of the flow data was determined as difference between respiration and cardiac spectral density: RmC_index = Respiration(R) – Cardiac(C). Further descriptions of all the parameters with schematic diagrams are given in Table 1.

**Table 1:** Description of flow parameters

**Parameter** **Unit**	**Description**	**Schematic Diagramm**
**Area** **mm**^**2**^	Area of ROI. Given is the temporal mean of all frames.	
**peakVelocity** **cm /sec**	The absolute maximum of the velocity-time-series obtained as the spatial mean of all velocity values within one ROI.	
**netFlow** **ml /min**	Temporal mean of the flow-time-series as obtained by multiplication of the mean velocity values and the corresponding ROI area.	
**stdFlow** **ml /min**	Standard deviation over all time points of the flow-time-series.	
**RmC_ Index**	Difference of respiration (R) and cardiac (C) spectral density. R represents the area under curve of the normalized spectrum from 0 to 0.5 Hz, while C refers to the 0.5 Hz interval covering the highest cardiac component close to 1 Hz. Thus, an RC_Index of 1 relates to respiration-modulated time curves and -1 to purely cardiac dependent dynamics. If both are balanced the RC_Index is zero.	

The ‘Area’ of each ROI and the flow parameters ‘peakVelocity’, ‘netFlow’, ‘stdFlow’, ‘RmC_index’ for all subjects were quantified and deployed for further statistical analysis.

### Statistical analysis

The subjects were initially divided into two groups: children (age 5–19 years, N = 24) and adults (age 22–40 years, N = 20). Linear regression analysis was performed to envision the dependency of flow parameters upon age in children and adult group separately. When there is significant linear change observed, the children group was further divided into three subgroups: group 1: 5–9 years (N = 7), group 2: 10–15 years (N = 6), group 3: 16–19 years (N = 11), and adult group was divided into two subgroups: group 4: 22–30 years (N = 14), group 5: 31–40 years (N = 6). ANOVA test was employed for the comparison between the subgroups. Tukey's multiple comparison test was applied for the comparison of paired groups. Statistical significance was determined as *p* < 0.05. In case of no significant linear change, independent t-test was performed between children and adult. All the statistical analyses were carried out using GraphPad Prism 9 program.

## Results

**Fig. 2:**
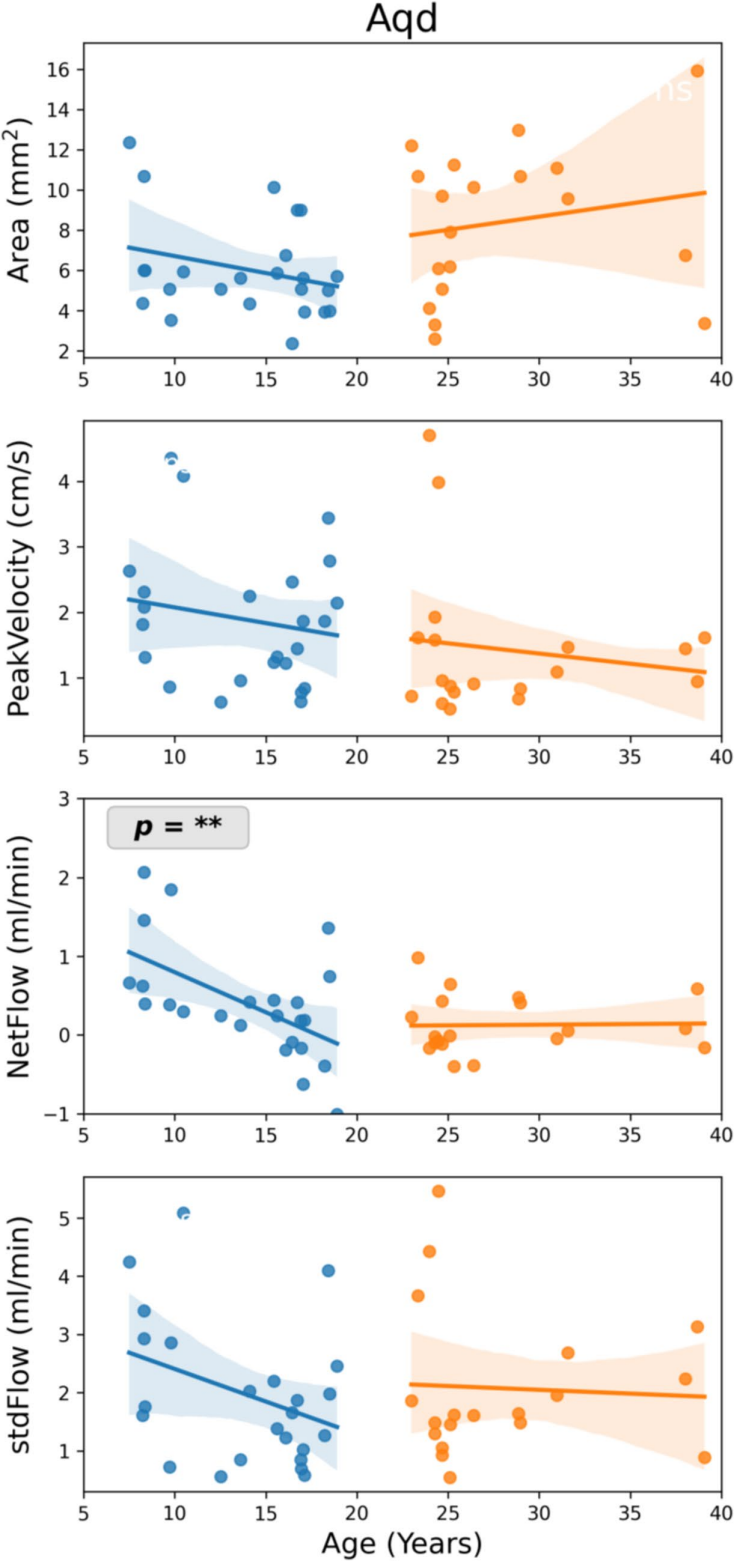
Linear regression between flow parameters and age at the aqueduct. Parameters are (from top to bottom): ROI Area (mm^2^), peakVelocity (cm/s), netFlow (ml/min), and stdFlow (ml/min). Stars indicate a slope significantly different from zero, i.e. *p* < 0.05*.* Shaded regions represent 95% confidence interval. Aqd = aqueduct, stdFlow = standard deviation over flow time series

**Fig. 3:**
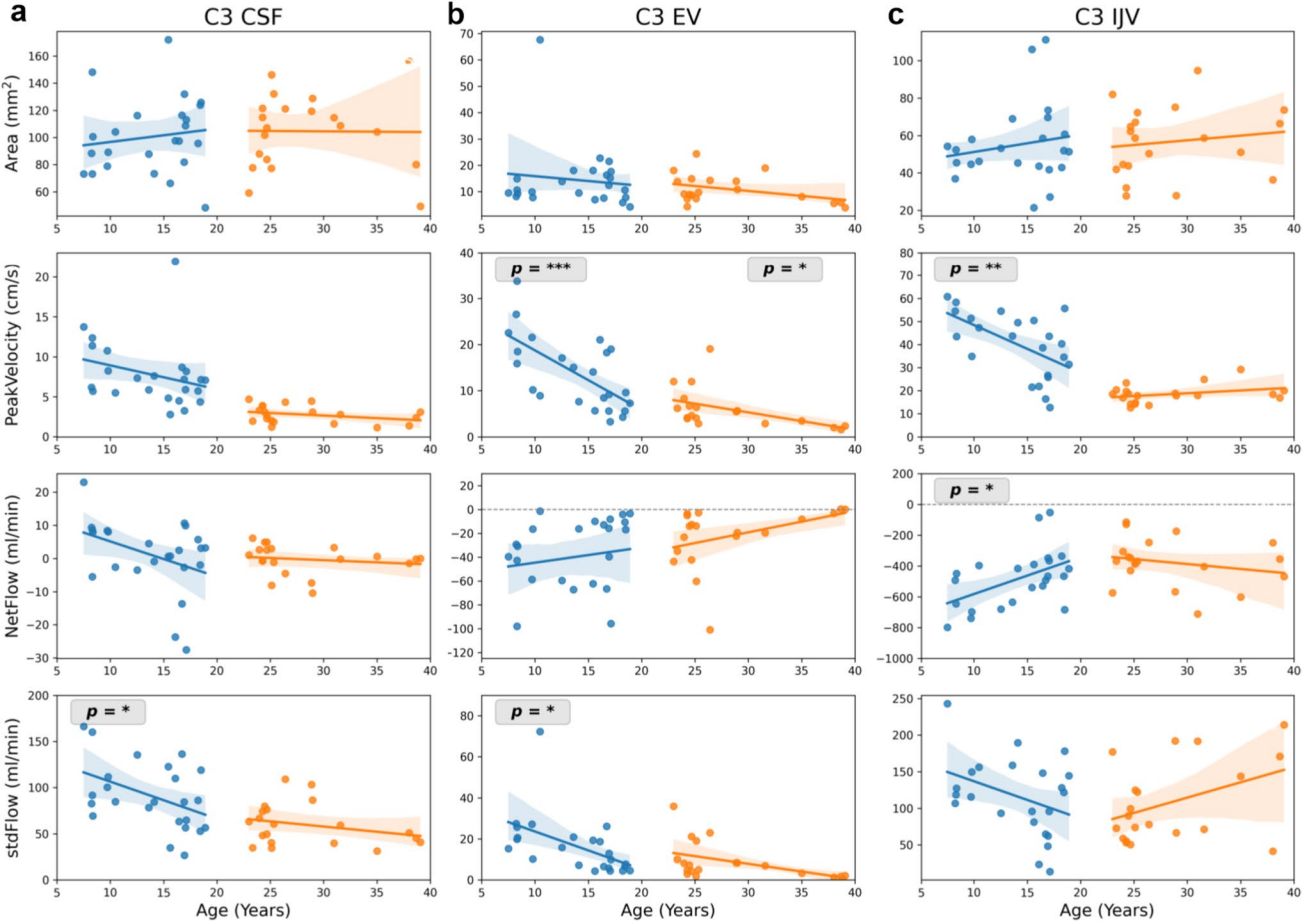
Linear regression between flow parameters and age at cervical spinal level C3. Parameters are (from top to bottom): ROI Area (mm^2^), peakVelocity (cm/s), netFlow (ml/min), and stdFlow (ml/min). ROIs from left to right C3 CSF (**a**), C3 EV (**b**), C3 IJV (**c**). CSF flow is shown in left column, venous flow in middle and right columns. Stars indicate a slope significantly different from zero, i.e. *p* < 0.05. Shaded regions represent 95% confidence interval. C3 = cervical spinal level 3, stdFlow = standard deviation over flow time series, EV = epidural veins, IJV = internal jugular vein

**Fig. 4:**
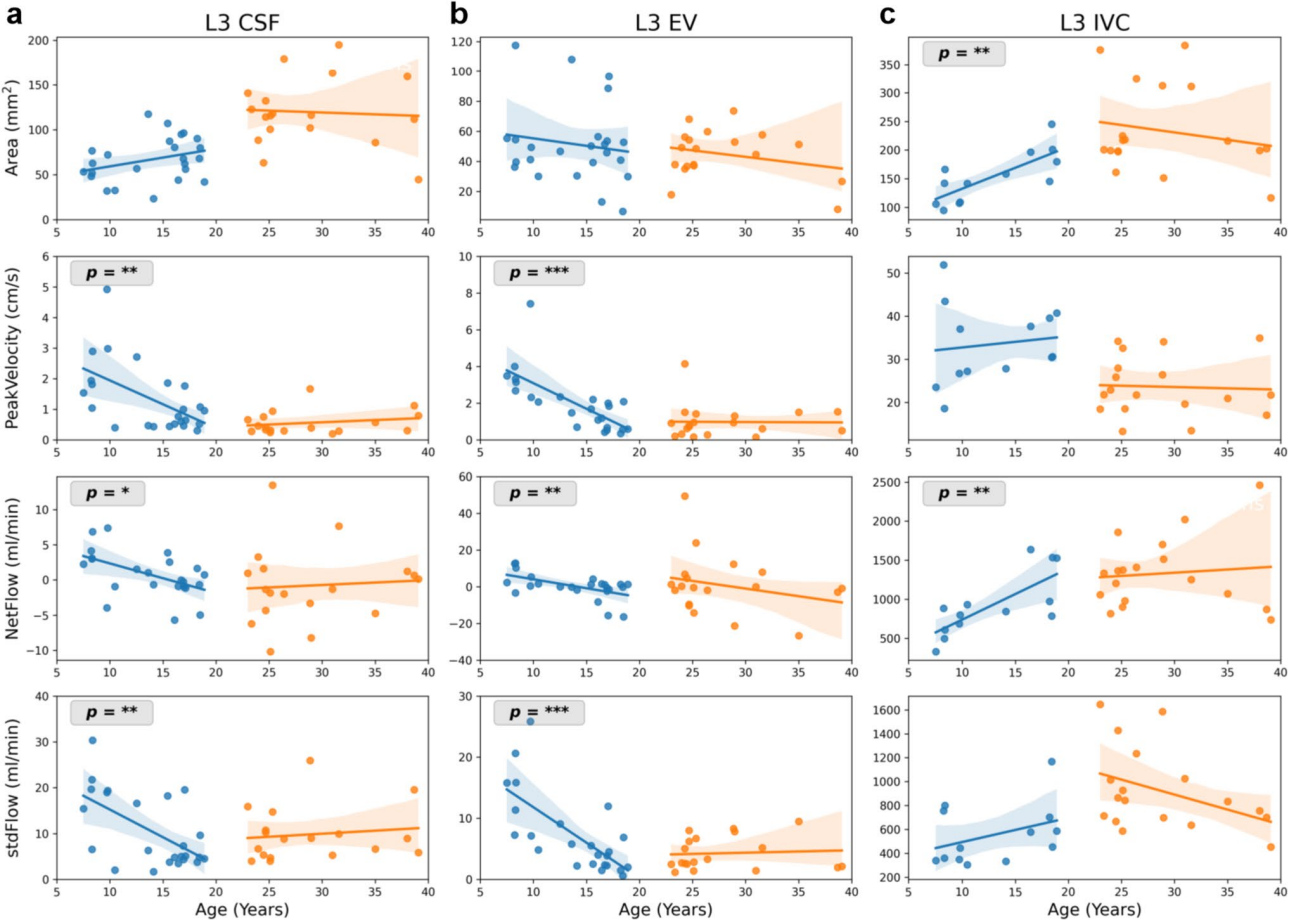
Linear regression between flow parameters and age at lumbar spinal level L3. Parameters are (from top to bottom): ROI Area (mm^2^), peakVelocity (cm/s), netFlow (ml/min), and stdFlow (ml/min). Stars indicate a slope significantly different from zero, i.e. *p* < 0.05. Shaded regions represent 95% confidence interval. CSF flow is shown in left column, venous flow in middle and right column. L3 = lumbar spinal level 3, stdFlow = standard deviation over flow time series, EV = epidural veins, IVC = inferior vena cava

Out of 44 participants flow measurements for IVC were not available in 13 subjects, and no flow signal could be detected in the IJV for 1 subject, in the Aqd for 1 subject and in the EV for 2 subjects. CSF and venous blood velocity over time at Aqd, C3 and L3 for three volunteers of different age are presented in supplement (Online Resource Fig. S1), to show the quality of the raw data and inter-subject variability. Figures 2, 3 and 4 depict linear regression analyses of the parameters Area, peakVelocity, netFlow and stdFlow with respect to age at Aqd, C3 and L3, respectively. Median value and range of 25%-75% of flow parameters for each age group and ANOVA results are given in Tables 2, 3 and 4.

**Table 2:** Aqueduct CSF flow – normal ranges and statistical analysis

**ROI**	**Parameters**	**Group-1** **5–9 yr** **(N = 7)**	**Group-2** **10–15 yr** **(N = 6)**	**Group-3** **16–19 yr** **(N = 11)**	**Group-4** **22–40 yr** **(N = 19)**	**ANOVA** **/Tukey**	**t-test**
**Aqd**	Area (mm^2^)		5.62 (4.36, 6.19)^a^		9.56 (5.58, 10.9)		*
	peakVelocity (cm/sec)		1.84 (1.16, 2.35)^a^		0.96 (0.81, 1.6)		ns
	netFlow (ml/min)	0.66 (0.51, 1.65)	0.27 (0.25, 0.39)	-0.09 (-0.29, 0.3)	-0.01 (-0.11, 0.42)	** 12,13,14	
	stdFlow (ml/min)		1.7 (0.98, 2.55)^a^		1.62 (1.37, 2.46)		ns

Median and range (25%, 75%). Tukey: Tukey’s multiple comparison test, two-digit number indicates the group pair those are significantly different from each other, Ex: '12' indicates that group-1 is significantly different from group-2

*ns* not significant, *ROI* region of interest, *yr *years, *N* number of subjects, *Aqd* Aqueduct

*p < 0.05; **p < 0.01; ***p < 0.001

^a^Group-1–3 were merged together and median was taken over age 5–19 years

**Table 3:** Cervical Spinal Level 3 - normal ranges and statistical analysis

**ROI**	**Parameters**	**Group-1** **5–9 yr** **(N = 7)**	**Group-2** **10–15 yr** **(N = 6)**	**Group-3** **16–19 yr** **(N = 11)**	**Group-4** **22–40 yr** **(N = 20)**	**ANOVA** **/Tukey**	**t-test**
**C3 CSF**	Area (mm^2^)		97.5 (81.1, 116.0)^a^		108.0 (83.0, 121.0)		ns
	peakVelocity (cm/sec)		7.1 (5.64, 8.35)^a^		2.75 (1.93, 3.45)		***
	netFlow (ml/min)		2.8 (-2.6, 8.13)		-0.06 (-1.23, 2.74)		ns
	stdFlow (ml/min)	100.0 (87.2, 136.0)	84.6 (79.7, 113.0)	64.8 (56.6, 98.1)	55.1 (40.6, 74.7)	** 14, 24	
**C3 EV**	Area (mm^2^)		10.6 (8.13, 16.7)^a^		9.16 (7.34, 14.2)		ns
	peakVelocity (cm/sec)	21.5 (17.2, 24.5)	11.5 (7.97, 14.8)	8.47 (5.63, 13.9)	4.62 (3.18, 6.58)	*** 12, 13, 14, 24	
	netFlow (ml/min)		-30.1 (-60.3, -12.4)^a^		-13.9 (-28.9, -4.17)		ns
	stdFlow (ml/min)	20.6 (17.5, 26.4)	17.6 (9.37, 20.5)	6.63 (5.16, 11.5)	6.89 (3.06, 9.22)	* 13,14	
**C3 IJV**	Area (mm^2^)		51.9 (44.2, 59.6)^a^		58.7 (43.3, 68.4)		ns
	peakVelocity (cm/sec)	52.9 (45.5, 57.4)	48.4 (44.6, 50.2)	31.3 (23.9, 39.4)	18.2 (16.4, 19.7)	*** 13, 14, 24, 34	
	netFlow (ml/min)	-671 (-729, -530)	-477 (-611, -400)	-417 (-479, -342)	-367.0 (-439, -291)	** 14,24	
	stdFlow (ml/min)		119.0 (87.2, 149.0)^a^		83.8 (64.5, 151.0)		ns

Median and range (25%, 75%). Tukey: Tukey’s multiple comparison test, two-digit number indicates the group pair those are significantly different from each other, Ex: '12' indicates that group-1 is significantly different from group-2

*ns *not significant, *ROI *region of interest, *EV *epidural vein, *IJV *internal jugular vein, *stdFlow *standard deviation of flow time series

*p < 0.05; **p < 0.01; ***p < 0.001

^a^Group-1–3 were merged together and median was taken over age 5–19 years

**Table 4:** Lumbar Spinal Level 3 - normal ranges and statistical analysis

**ROI**	**Parameters**	**Group-1** **5–9 yr** **(N = 7)**	**Group-2** **10–15 yr** **(N = 6)**	**Group-3** **16–19 yr** **(N = 11)**	**Group-4** **22–40 yr** **(N = 18)**	**ANOVA** **/Tukey**	**t-test**
**L3 CSF**	Area (mm^2^)		65.2 (51.0, 82.1)^a^		116.0 (101.0, 139.0)		***
	peakVelocity (cm/sec)	1.94 (1.68, 2.93)	0.45 (0.43, 1.51)	0.64 (0.52, 0.98)	0.4 (0.29, 0.73)	*** 13,14	
	netFlow (ml/min)	3.07 (2.62, 5.48)	1.28 (-0.23, 2.3)	-0.88 (-1.07, -0.14)	-1.34 (-4.09, 1.15)	* 13,14	
	stdFlow (ml/min)	19.4 (17.2, 20.7)	5.05 (2.45, 14.0)	4.81 (4.42, 6.19)	8.89 (5.47, 10.6)	** 12,13,14,34	
**L3 EV**	Area (mm^2^)		48.0 (38.5, 54.7)^a^		48.5 (36.9, 55.2)		ns
	peakVelocity (cm/sec)	3.32 (2.91, 3.74)	1.87 (1.52, 2.17)	0.65 (0.54, 1.51)	0.77 (0.41, 1.36)	*** 12,13,14,23,24	
	netFlow (ml/min)	5.44 (1.39, 11.5)	0.66 (-0.07, 1.57)	-0.41 (-5.25, 1.24)	-0.36 (-6.29, 5.71)	* 13	
	stdFlow (ml/min)	15.8 (9.32, 18.2)	5.18 (3.11, 5.7)	2.34 (1.75, 4.25)	2.83 (2.31, 6.45)	** 12,13,14	
**L3 IVC**	Area (mm^2^)	108 (106, 134)	150 (146, 154)	197 (180, 201)	209.0 (199.0, 290.0)	** 12,13,14	
	peakVelocity (cm/sec)		30.6 (27.3, 39.6)^a^		21.8 (18.8, 27.6)		***
	netFlow (ml/min)	648 (524, 772)	888 (867, 910)	1529 (975, 1535)	1293 (1000, 1488)	*** 12,13,14,34	
	stdFlow (ml/min)		454.0 (349, 702)^a^		454.0 (349, 702)		ns
	N^b^	6	2	5	18		

Median and range (25%, 75%). Tukey: Tukey’s multiple comparison test, two-digit number indicates the group pair those are significantly different from each other, Ex: '12' indicates that group-1 is significantly different from group-2

*ns* not significant, *ROI *region of interest, *EV* epidural vein, *IVC *inferior vena cava, *stdFlow *standard deviation of flow time series

*p < 0.05; **p < 0.01; ***p < 0.001

^a^Group-1–3 were merged together and median was taken over age 5–19 years

^b^N: number of subjects where IVC measurements were obtained

In adult volunteers, none of the parameters revealed a significant variation with respect to age in linear regression analysis (Figs. 2, 3 and 4), except peakVelocity of epidural venous flow at C3 (Fig. 3). Henceforth, all volunteers in age range 22–40 years were combined to one group (group-4) and used in ANOVA analysis.

### CSF flow dynamics at Aqd, C3 and L3

In children and adolescents (age 5–19 years) CSF flow depicts a decreasing trend according to age. Notably, in linear regression analysis, significant age-related changes were observed for netFlow at Aqd (Fig. 2), stdFlow of CSF at C3 (Fig. 3a), and peakVelocity, netFlow and stdFlow of CSF at L3 (Fig. 4a). ANOVA analysis demonstrated significant differences among the groups-1–4. Tukey’s multiple comparison revealed that the youngest group (group-1) is significantly different than the other groups (Tables 2, 3 and 4). Area of Aqd and L3 CSF significantly increased from children to adults while no change of area of C3 CSF was found in t-test. PeakVelocity of CSF at Aqd appeared to be independent of age, while these values at C3 significantly decreased from children to adults.

### Venous flow dynamics at C3 and L3

Area of EV and IJV did not alter with age, while area of IVC significantly increased with age. ANOVA depicted a significant difference in area of IVC between age groups (Table 4). Epidural venous flow parameters at C3 and L3 reflect similar decreasing trend with age as CSF in children. Particularly, peakVelocity and stdFlow of C3 EV (Fig. 3b) and L3 EV (Fig. 4b) significantly decreased with age. Blood flow in major vessels as C3 IJV, net downward flow and peakVelocity decreased (Fig. 3c), while at L3 IVC net upward flow increased with respect to age (Fig. 4c).

#### Respiration and cardiac components of CSF dynamics

RmC_index showed no significant change according to age at all CSF locations. The mean RmC_index across all subjects (with 95% CI) at different locations is shown in supplement (Online Resource Fig. S2). Here, a negative RmC_index indicates that cardiac modulations dominate CSF and venous flow dynamics at C3. Positive RmC_index at L3 hint at flow dynamics predominantly driven by respiration. For CSF flow in the Aqd neither a respiratory nor cardiac dominance on the flow dynamics is observed (equal contribution), as indicated by RmC_index close to zero.

## Discussion

Subjects ranging from early childhood to adulthood were included in this study covering critical stages of physical development. Along with CSF flow in Aqd and spinal canal venous flow dynamics were analyzed. Flow parameters such as netFlow, stdFlow, peakVelocity, and area were determined and normal ranges for four different age groups are provided. Our results demonstrate that physical growth during childhood influences characteristics of CSF and venous blood flow, while no changes were observed during adulthood. Therefore, evaluations of flow dynamics for individual pediatric patients during their clinical management, require careful consideration of parameters, locations and age and concomitant growth-related changes.

### Cerebral aqueduct

Aqd areas could be an important parameter for assessment of an aqueductal stenosis. Here, Aqd sizes revealed an age-dependent increase not from childhood to adolescence, but in the adult age groups. During free breathing no consistent temporal variations of ROI areas occurred. Henceforth, we consider the observed ROI changes over age a reflection of physical growth. Consistent with our results a study by Öztürk et al. in pediatric subjects covering an age range of 1 month–17 years found no difference in Aqd area [13]. Recent studies of the human brain within the entire lifespan reported exponentially growing CSF spaces from the beginning of the fourth decade on [27]. Moreover, in 20–65 years-old volunteers aqueduct sizes increased significantly with age in line with our results. This indicates a slow and gradual change in Aqd area which only could be visible in a wider age range. Nevertheless, although the trend observed here agrees with the literature, actual numbers for the Aqd areas differ. A direct comparison of values from separate studies should be drawn with caution as they depend upon imaging sequence, scanner type and post-processing methods.

NetFlow in the Aqd decreased from childhood to adolescence, a period defined by pronounced physical growth and brain development. Interestingly, the positive netFlow rates in the young children point towards effective CSF movement from the fourth to the third ventricle. NetFlow significantly shifts to more balanced dynamics with growth into adolescence and adulthood. This observation is in line with a study by Öztürk et al. which reports more flow in cranial direction than caudally in children age 1–10 years [13]. Similar results were also described in previous cardiac gated PC MRI studies of healthy children, where in young children the aqueduct flow is directed upwards into the ventricular system [28].

PeakVelocity has been demonstrated by cine PC-MRI to decrease significantly from childhood to adolescent [14]. Our data reflect a decreasing trend in aqueductal peakVelocity according to age albeit without reaching statistical significance. The small sample size might be one explanation. Another reason could be the imaging protocol as most of the previous studies use cine PC-MRI where many cardiac cycles are averaged minimizing noise. In contrast, the RT-PC MRI peak velocity may be susceptible to a single high or low velocity value, as averaging over the small aqueductal area does not preclude such effect.

### Cervical spinal level 3 (C3)

At spinal level C3 the measurement protocol not only focusses on the subarachnoid CSF space, but also on venous flow in intraspinal EV as well as on IJV in the neck outside the bony spinal canal. The flow parameters at C3 affected by age are peakVelocity and stdFlow or in other words flow pulsatility which demonstrate a significant reduction both for CSF and venous flow. Since the areas of all three ROIs exhibited no significant age dependency, the change in pulsatility could be associated with heart pulsations at this localization, which are reported to decrease from children to adults [29]. In support of this argument, the RmC_index indicates cardiac dominance in the flow measurements at level C3. Studies on venous dynamics in the age groups investigated here are extremely scarce besides they often used other modalities with different protocols precluding direct comparisons [30]. Aging effects of blood and CSF flow in young vs elderly healthy adult subjects i.e. a decrease of mean jugular venous flow have been reported probably more in line with our results [15] although further studies are warranted.

Although netFlow also appears to be decreasing in CSF and EV, however, only IJV netFlow reaches significance. The overall variability of netFlow is consistent with other studies of healthy subjects [31, 32]. Of note, other parameters of the brain’s blood supply, such as cerebral blood flow (CBF), demonstrate similar trends during childhood. A study applying arterial spin labeling revealed a significant decrease of CBF between about 4 and 15–20 years in gray and white matter [33]. Similar observations have been published earlier with perfusion computed tomography. Here, global average values of cerebral blood volumes decreased from 2 to 8 years of age [34].

### Lumbar spinal level 3 (L3)

Spinal level L3 was included in adherence to our study protocol designed in view of possible clinical applications encompassing CSF dynamics of brain and spinal canal as well as of intra- and extraspinal venous flow. At L3, the parameters for both CSF and epidural venous flow are largely age dependent. Compatible with physical growth, ROI areas of subarachnoid CSF space and IVC increased significantly from children to adults. The alterations of CSF and EV dynamics over the age groups resemble those at C3. At L3 both pulsatility and netFlow are affected. Venous netFlow in intraspinal EV demonstrate similar age relations as CSF flow. Flow dynamics are primarily driven by respiration, as indicated by the positive RmC_index. It is reported that respiration rates decrease with age, particularly between 1st and 20th year of life [35, 36]. On the other hand, lung capacity and breathing volume increase continually over that time [37]. Because breathing governs intra-thoracic and intra-abdominal pressures, it can be assumed that respective physiological changes contribute to the evolution of flow dynamics over age.

In contrast to CSF and EV, netFlow in IVC reveals a significant increase with age. Being the largest venous vessel, collecting blood from the whole lower body, capacity and flow of IVC rise with physical growth. Moreover, in this major vein the increase of area with age parallels the enhancement of netFlow. Most probably these observations are related to the well documented developmental changes of circulatory and respiratory parameters including increase of blood pressure, decrease of pulse and respiratory rates. More detailed hypotheses about the exact underlying mechanisms are, however, beyond the scope of this study.

### Limitations

The comparatively small number of subjects considering the wide range of age might represent a limitation. Children below five years of age could not be considered due to ethical reasons as MRI examinations would require sedation. In addition, not all age groups comprise equal numbers of subjects. It is likely, that a larger study population may reveal further significant differences that currently could not be recognized. As in a potential clinical setting, we did not control for caffeine consumption by the participants or time of scan during the day. These aspects have been shown to influence CSF production, circulation and CBF [38, 39]. Furthermore, we refrained from a separate analysis for male and female subjects in this cohort. Discrepant results have been reported by previous studies with respect to gender dependency of CSF flow. Oner et al. and Unal et al. found no statistically significant difference comparing aqueductal flow between males and females [12, 14]. In contrast, according to Schmid Daners et al., aqueductal but not spinal CSF mean flow exhibited significant gender-dependent variations among young adults [40].

## Conclusions

In preparation for a broader clinical application, we have established normal ranges for relevant CSF and venous flow parameters using RT-PC MRI. These flow parameters exhibit notable age dependency that varies with anatomical location. Therefore, it is imperative to take the patient's age into account, particularly in pediatric cases, when interpreting the flow parameters. RT-PC MRI results were consistent with physiological dynamic changes reported in the literature. The high spatio-temporal resolution, independence of periodic trigger and robustness against movement artefacts renders it a suitable tool for broader clinical utilization. Its application in clinical care will contribute to a better understanding of CSF flow pathologies in individual patients and thus improve their management. On a broader scale it might shed light into pathophysiological processes of still enigmatic hydrocephalus forms and lead to more specific therapies.

### Supplementary information

Below is the link to the electronic supplementary material.

Supplementary file1 (DOCX 1135 KB)

Supplementary file2 (DOCX 442 KB)

## Data Availability

The datasets used and/or analyzed during the study are available from the corresponding author on reasonable request.

## Declarations

### Disclaimer

JF is a co-inventor of a patent describing the real-time MRI method used here.
