# Supplementary material for: CSF and venous blood flow from childhood to adulthood studied by real-time phase-contrast MRI
Source: Childs Nerv Syst. 2024 Jan 11;40(5):1377–88. doi: 10.1007/s00381-024-06275-1 (PMC11026278; doi:10.1007/s00381-024-06275-1)
Supplement: Supplementary file 1 — Supplementary file1 (DOCX 1135 KB) [file 381_2024_6275_MOESM1_ESM.docx]

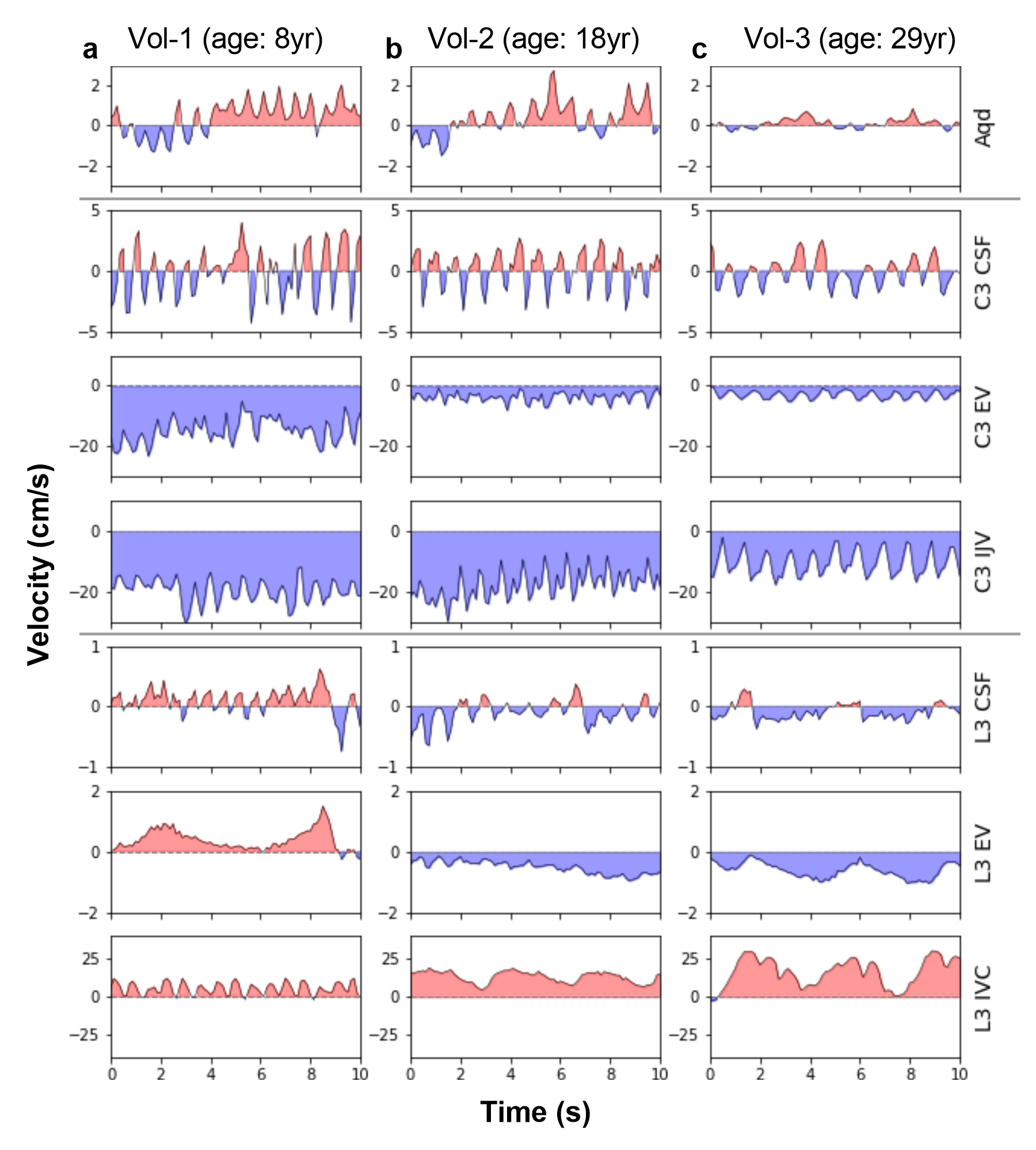


**Fig. S1: Velocity time measurements of all location**.

Flow time curves from Aqd, C3 CSF, C3 EV, C3 IJV, L3 CSF, L3 EV, L3 IVC (top to bottom row) of three volunteers of different age. (a) Volunteer-1 age 8 years, (b) volunteer-2 age 18 years and (c) volunteer-3 age 29 years. Red color indicates upward flow and blue color indicates downward flow. Aqd = aqueduct, C3 = cervical spinal level 3, L3 = lumbar spinal level 3, EV = epidural veins, IJV = internal jugular vein, IVC = inferior vena cava, yr = years.
