# Supplementary material for: CSF and venous blood flow from childhood to adulthood studied by real-time phase-contrast MRI
Source: Childs Nerv Syst. 2024 Jan 11;40(5):1377–88. doi: 10.1007/s00381-024-06275-1 (PMC11026278; doi:10.1007/s00381-024-06275-1)
Supplement: Supplementary file 2 — Supplementary file2 (DOCX 442 KB) [file 381_2024_6275_MOESM2_ESM.docx]

**
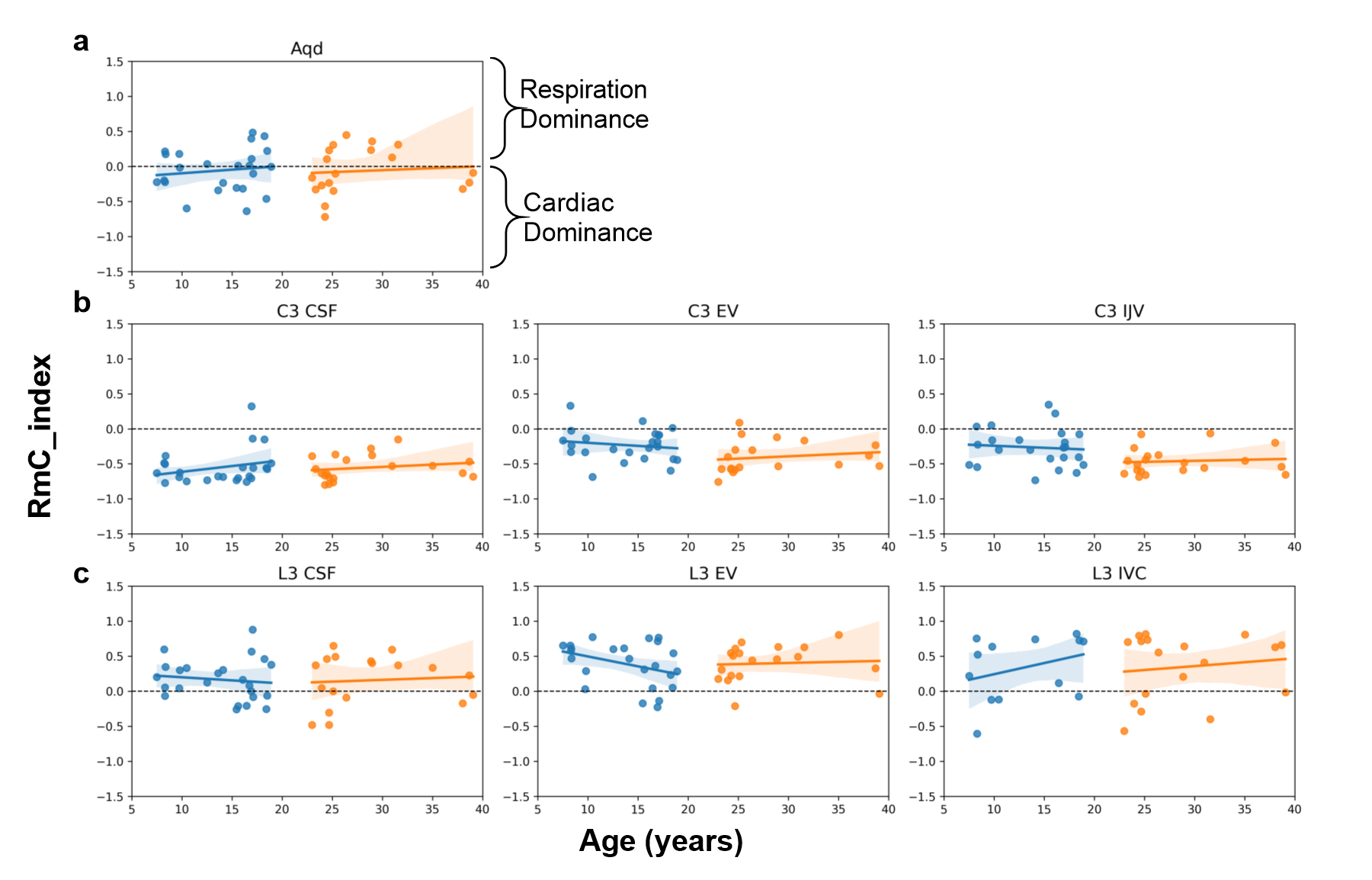
**

**Fig. S2: Respiratory vs cardiac components of CSF and venous flow with age**

RmC_index describes difference between respiratory and cardiac spectral density of flow time curve across all subjects for normal breathing. (a) In Aqd cardiac and respiration components contribute approximately equally to CSF flow. (b) Negative values indicate dominance of cardiac modulations at C3 and (c) positive values of respiratory modulations at L3. Shaded regions represent 95% confidence interval. RmC_index = Respiration–Cardiac spectral density, Aqd = aqueduct, C3 = spinal level C3, L3 = spinal level L3, EV = epidural veins, IJV = internal jugular vein, IVC = inferior vena cava.
